# On Mr. Adams's New Operation for the Cure of Cataract

**Published:** 1814-04

**Authors:** 


*For the Medical and Physical Journal.*

### *On Mr. Adams's new Operation for the Cure of Cataract.*

IN the last month's Number of your Journal, it was stated that Mr. Adams had lately, by a new operation for the cure of cataract, been very successful in several cases which had before received no benefit under the late oculists of Greenwich Hospital; and, in the Courier of the 17th inst. the public were informed that —— Turnbull, esq. had been restored to sight in five days, by a "new method of extracting the cataract, by Mr. Adams, the oculist."

The information contained in your pages, might, perhaps, be a sufficient invitation to Mr. A. to make known the particular points which tend to give him such superiority of success; the public good and the happiness of individuals afflicted by darkness, must be claims on his diligence to delay as little as possible the promulgation of his improvements in this department of surgery.

I have had many opportunities of witnessing the progress of the absorption of cataracts when *cut up* as recommended by Mr. A. and though, in most instances, the process is soon completed, (not generally requiring more than two operations), yet I have also seen its dissolution extremely slow and tedious to patients who, on some occasions, have abandoned all thought of a repetition of an operation from which they have received but limited and procrastinated advantage. I am a follower of the operations practised by Mr. Adams, and am fully convinced of their superiority to those formerly recommended. The cataracts of old persons, which, in colour and consistence, often resemble horn, though admitting of a removal by repeated operations, are too frequently slow and difficult of absorption, and I conceive a safe method of extracting them becomes a desirable object; but such a method should bear the form of improvement on the former practice. In reading the notices before spoken of, the words "new" mode of "extraction" caught my attention, and I felt a hope that it might tend to remove the stumbling blocks which lie in the way of the blind in their road to light.

I, perhaps,

I, perhaps, may not be quite correct in having made the medical news of a public paper a subject for your valuable pages; but, concluding that Mr. Turnbull's case was made public by those who were acquainted with it, I feel it a point of importance that the profession should early become acquainted with the *new mode of extraction.*

*Feb.* 26, 1814. CHIRURGUS OCULARIUS.

